# Engaging men in an mHealth approach to support postpartum family planning among couples in Kenya: a qualitative study

**DOI:** 10.1186/s12978-019-0669-x

**Published:** 2019-02-11

**Authors:** Elizabeth K. Harrington, Erin E. McCoy, Alison L. Drake, Daniel Matemo, Grace John-Stewart, John Kinuthia, Jennifer A. Unger

**Affiliations:** 1 Department of Obstetrics and Gynecology, University of Washington, 1959 NE Pacific St., Boxes 356460, Seattle, WA 98195 USA; 2 Department of Global Health, University of Washington, Harborview Medical Center, 325 Ninth Ave., Boxes 359909, Seattle, WA 98104 USA; 3 Department of Obstetrics & Gynecology, University of Nairobi, Nairobi, Kenya; 4 Departments of Global Health, Medicine, Epidemiology and Pediatrics, University of Washington, Harborview Medical Center, 325 Ninth Ave., Boxes 359909, Seattle, WA 98104 USA; 5 Department of Research & Programs, Kenyatta National Hospital, PO Box 20723-00202, Nairobi, Kenya; 6 Department of Obstetrics and Gynecology, University of Washington, Harborview Medical Center, 325 Ninth Ave., Boxes 359909, Seattle, WA 98104 USA

**Keywords:** Kenya, Postpartum contraception, Family planning, mHealth, SMS, Innovation, Couples, Men and family planning, Qualitative

## Abstract

**Background:**

Involving male partners in family planning (FP) education and counseling may improve FP utilization and help meet couples’ reproductive health needs in the postpartum period. We aimed to explore Kenyan men’s and women’s perspectives on an interactive short message service (SMS) approach to support postpartum FP decision-making, and inform intervention content for a randomized controlled trial (RCT).

**Methods:**

We conducted four focus group discussions (FGD) among men (*n* = 35) and two among pregnant/postpartum women (*n* = 15) in western Kenya. Female participants were recruited at antenatal clinics; male participants were referred by antenatal attendees. FGDs included participant critique of pilot theory-based SMS messages. FGD transcripts were coded by two investigators and analyzed using an iterative, modified grounded theory approach. These data informed the intervention and RCT design, in which women had the option to refer male partners for trial enrollment.

**Results:**

Men strongly desired inclusion in FP programs, and frequently discussed negative relationship consequences of women’s covert contraceptive use. Female and male participants voiced a variety of concerns about contraceptive side effects and potential harms, which were central to narratives of community influence on personal contraceptive choices. Most participants felt that receiving FP-focused SMS and including men would be beneficial. They perceived that SMS dialogue with a nurse about FP could reduce misperceptions and may stimulate communication within couples, thereby improving contraceptive access and continuation. Shared decision-making around FP within couple relationships, in consultation with clinicians, was highly valued.

**Conclusions:**

Health concerns about FP and limited couple communication are perceived contributors to postpartum unmet contraceptive need. With women’s consent, the inclusion of male partners in FP services, and specifically in an mHealth SMS intervention, is acceptable and desired. Receiving SMS may trigger communication about postpartum FP within couples. SMS content should address contraceptive knowledge gaps, anticipated side effects and FP misperceptions, and allow for real-time method choice assistance.

## Plain English summary

Many women in low-income countries who desire to prevent pregnancy in the postpartum period (after a birth) face barriers in accessing contraception. Mobile health (mHealth) strategies have the potential to reach both male and female members of a couple with family planning (FP) counseling. The purpose of this study was to understand Kenyan men’s and women’s views on the use of two-way SMS with a health provider to support postpartum contraceptive use, which would inform an mHealth intervention tailored for couples. We conducted six focus group discussions with a total of 50 Kenyan men and women, and qualitatively analyzed the data to identify themes in participants’ perspectives. Participants identified a lack of FP knowledge among men, contributing to negative views of FP in the community. Male partner involvement was viewed as acceptable and desired in mHealth FP programs using SMS, and few women voiced privacy concerns about including men. Men and women perceived that engaging couples in FP counseling via SMS would help overcome barriers to postpartum contraceptive use: by reducing misperceptions about contraceptive harms, providing information about potential side effects, and encouraging communication within couples. SMS content for an intervention should address FP knowledge gaps and anticipated side effects. Participants desired the opportunity for SMS dialogue with a nurse in order to allow for real-time guidance on contraceptive method choice. These findings were used to design a novel two-way SMS program to improve postpartum contraceptive access in Kenya.

## Background

Strengthening family planning (FP) programs and health services for postpartum women is increasingly recognized as a global sexual and reproductive health (SRH) priority [1–3]. Despite general desire to space and limit pregnancies after birth and the evidence for maternal and child health benefits of postpartum FP [4], data from many low- and middle-income countries demonstrate that contraceptive use and continuation among postpartum women are low—leaving women at risk for unintended and short interval pregnancies [5]. In Kenya, unmet need for contraception among postpartum women within the first 23 months postpartum has been estimated at 57%, with 50% of interpregnancy intervals less than the WHO-recommended 2 years [6].

Multiple health systems and socio-cultural barriers to postpartum FP use have been described, from concerns about side effects and safety of modern FP methods [7, 8], low risk perception of pregnancy due to lactation and lack of menses [3, 9], concern for partner disapproval [10], and poor postpartum visit attendance with inadequate counseling in the clinical setting [11, 12]. Innovative strategies to overcome these barriers and address women’s postpartum FP needs are urgently needed.

Researchers and policy organizations have emphasized the critical need to engage men and couples in FP programming since the 1994 International Conference on Population and Development [13–15]. While studies show an association between male involvement and contraceptive use in some settings [16, 17], few interventions have incorporated men. Findings from qualitative work in Kenya show that many men and women desire male inclusion in FP education and decision-making, and that FP-related stigma, men’s concerns about side effects, and shifting gender relations contribute to male resistance [18, 19]. However, best practices in harnessing male involvement have not been demonstrated.

Mobile health (mHealth) technologies, specifically short message service (SMS) programs, have shown benefit in various SRH contexts in resource-limited settings [20–22]. However, few mHealth interventions have focused on supporting postpartum contraceptive use or including male partners or couples. Additionally, while evaluation of users’ experience with mHealth programs is critical to successful implementation [23], details on development of SMS content for SRH is lacking, particularly end-user or community perspectives on which messaging strategies are anticipated to be most acceptable, desired, and effective [24, 25].

**Fig. 1:**
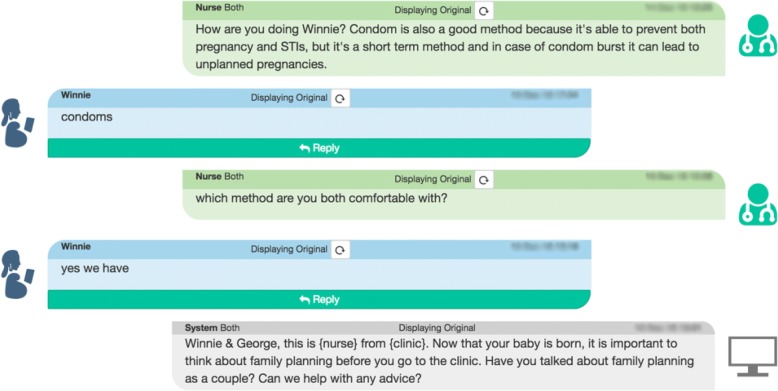
Screenshot of an example interaction in the Mobile WACh platform (read bottom to top). The system sends an automated message (grey) specific to the participant’s estimated/delivery date, and the participant may respond free of charge (blue). A nurse responds (green)

The Mobile WACh mHealth platform is a human-computer hybrid communication system originally designed for a maternal child health intervention in Kenya [26]. The system allows for both automated sending of tailored health-related SMS messages and two-way SMS interaction between participants and a health care provider (Fig. 1). We sought to adapt this platform to create an interactive SMS intervention supporting both women and couples in postpartum contraceptive decision-making, method initiation, and method continuation. The intervention would provide FP education and real-time clinician counseling, reminders, and encouragement—as well as promoting male involvement in FP decision-making when desired and appropriate.

The purpose of this study was to explore men’s and women’s perspectives on using SMS to facilitate postpartum FP counseling in Kenya and engage men in FP decision-making. Drawing on qualitative data from focus groups, we aimed to describe men’s and women’s views on receiving FP-related information via SMS, to guide an approach to messaging and content development, and to consider how receiving FP-related SMS may affect FP decision-making and communication between partners.

## Methods

### Participants

This study was conducted at Government of Kenya hospitals located in two counties in the Nyanza region of western Kenya. These hospitals serve a primarily low- to middle-income rural population, the majority of whom identify with the Luo ethnic group. Our sampling frame sought to include men and women who reflected the eligibility criteria of the planned RCT. Women were 14 or older, currently pregnant or within 6 months postpartum, and HIV negative by self-report; pregnant women and mothers younger than 18 are considered emancipated consenting adults under Kenyan law. Male participants had a pregnant female partner and were 18 or older. All participants had daily access to a mobile phone, were literate in Dholuo, Kiswahili, or English, and were willing to provide written informed consent. Female participants were recruited in the antenatal clinic waiting area by study staff, and interested women were screened for eligibility. Eligible women who desired to participate in the FGD were given a date/time and provided contact information. Study staff asked women who had not been recruited themselves if they would consider referring male partners to the study. Contact information was obtained for referred men, and the male FGD facilitator screened and scheduled potential participants by phone.

The Institutional Review Boards at the Kenyatta National Hospital and the University of Washington provided ethical approval for the study. We also received approval from the Ministry of Health at the facility level.

### Data collection

A total of six FGDs were conducted from April to June 2016, four among men (*n* = 35) and two among women (*n* = 15). Two facilitators with qualitative research experience, fluent in Dholuo and English, moderated the FGDs using a sex-specific semi-structured guide; the men’s groups were facilitated by a man, and the women’s groups by a woman. Facilitators had no connection to the provision of clinical care at the health facilities. Each FGD was conducted in a private clinic area on a weekend-day, lasted approximately 90 min, and included 7–11 participants. In addition to the facilitator, a study note taker was present. Prior to FGD initiation, study nurses obtained written informed consent and administered a brief survey capturing basic socio-demographic information. All FGDs were digitally audio-recorded with participant consent. Participants received 400 Kenyan Shillings (approximately 4 US dollars) for transportation reimbursement.

Ten sample SMS messages were available in English, Kiswahili, and Dholuo for review by FGD participants (Table 2). Each automated message is personalized, designed to provide actionable information, and ends with a question or statement to engage the participant. Feedback on content and structure of the sample SMS messages was elicited at the end of each FGD.

#### Data analysis

Interviews were transcribed verbatim in Dholuo and translated into English by FGD facilitators. Transcripts were uploaded into Dedoose 7.0.23 (SocioCultural Research Consultants, LLC 2016). EKH read through the raw data and created a list of initial codes to represent a priori and new concepts of interest. EKH and EEM coded the transcripts from both female FGDs and 2 male FGDs using this initial codebook, proposing new codes in real-time through an open coding process (i.e., codes suggested by textual content). The codebook was revised after discussion and consensus. All transcripts were then re-coded using the revised codebook, and investigators met to discuss how codes were used and text interpreted. New codes were added as thematic elements emerged, and were incorporated into the transcripts in additional iterations of coding. Thematic memos were developed, condensing quotations into analytic text. This inductive, iterative analytic process was adapted from the constructs of Grounded Theory [27]. However, in departure from classic Grounded Theory methods, a less interpretivist approach was taken, with specific a priori research questions. Data were collected and analyzed asynchronously due to logistical constraints. [28]

### Theoretical framework for SMS development (Fig. 2)

The initial intervention design and SMS messages were based on behavioral theory and experience with two previous Mobile WACh interventions [29]. The Theory of Planned Behavior [30] was used to guide SMS message development. We propose that educational and encouraging FP-related two-way messaging could affect attitudes and beliefs about contraception and its use, which may alter one’s perception of risks and benefits and subsequently the intention to use FP. The construct of “perceived behavioral control,” referring to one’s perceived ability to take action with respect to a behavior, is a key component of both the intention to use FP and FP initiation/continuation after delivery. Messaging may challenge personal subjective and social norms about postpartum pregnancy risk and FP use, though perhaps in conflict with community perceptions or codes of behavior. These social norms also have the potential to change one’s intentions or perceived control over FP use. Couple communication around FP as a result of SMS, as well as counseling about method choice and anticipatory guidance about side effects, may affect perceived behavioral control among men and women.

## Results

Participant characteristics are summarized in Table 1. Male participants (*n* = 35) had a median age of 30, and 97% were married with median relationship duration of 5 years. The majority (60%) had at least some secondary school education, and most (60%) reported prior personal or partner use of a FP method. Women’s (*n* = 15) median age was 25, and 87% were married with a 2-year median relationship duration. Most female participants (60%) had a primary school education or less, had at least one child (80%), and 53% had any FP experience.

**Table 1:** Participant characteristics

*Characteristic*	*Men (n = 35)* *n*(%) / median (IQR)		*Women (n = 15)* *n*(%) / median (IQR)	
Age (completed years)	30	(25–36)	25	(19.5–27.5)
Partnered	35	(100%)	14	(93%)
Married	34	(97%)	13	(87%)
Pregnant	–		6	(40%)
Relationship duration (years)	5	(2–10)	2	(1–9)
Education				
Primary or less	14	(40%)	9	(60%)
Secondary (some/completed)	11	(31%)	4	(27%)
Above secondary	10	(29%)	2	(13%)
Share phone with partner	7	(20%)	6	(40%)
Number of pregnancies	–		2	(1–3)
Number of children				
0	7	(20%)	3	(20%)
1–2	20	(57%)	9	(60%)
3 or more	8	(23%)	3	(20%)
Ever use of FP^a^	21	(60%)	8	(53%)
Type of FP used^a,b^				
Male condom	11	(31%)	3	(20%)
Pill	2	(6%)	2	(13%)
Injection	8	(23%)	5	(33%)
Implant	7	(20%)	2	(13%)

^a^Includes personal method use and partner use

^b^Participants could identify > 1 method

**Fig. 2:**
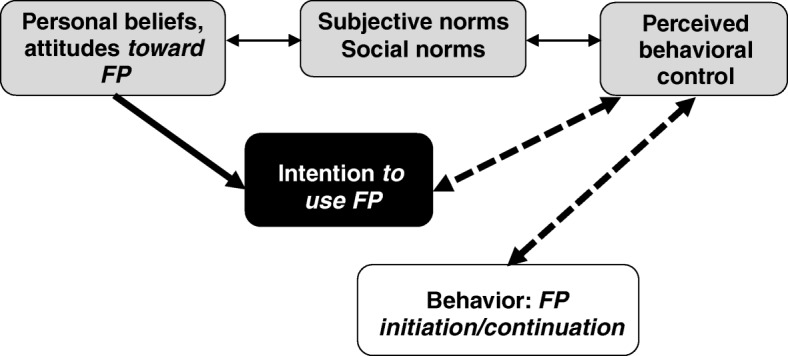
Schematic of the theoretical framework for SMS development, adapted from the Theory of Planned Behavior [30]

### Men strongly desire inclusion in FP decision-making

Male participants expressed an overwhelming preference for—and sense of entitlement to—inclusion in FP decision-making. Yet, the majority of male participants alluded to men’s general lack of knowledge about FP, and commented on what they felt were the most appropriate sources of FP education. Community education campaigns, presentations at chiefs’ *barazas* (assembly of village leaders) and churches, radio messaging, and home visits were the most frequently recommended strategies for educating men. In general, female partners were not considered ideal, or even trustworthy, sources.

*“As a man, the wife brings you positive information about FP, but you as the husband, you just dwell on the negative things and you are against it because you have no information.” (20 year-old man, no children).*

Men perceived that the imbalance of FP knowledge between men and women not only contributes to community FP stigma, but negatively affects couple communication:

*“This problem comes when somebody has idea while the other person is totally green about FP, so starting the conversation is a challenge. I think a lot of awareness should be done so that both can have some information.” (28 year-old man, no children).*

In all FGDs, often without prompting, men universally noted the necessity of “consultation”: that is, that the female partner should discuss FP with the male partner prior to method initiation. Personal and community narratives of women’s covert contraceptive use and its consequences figured prominently in the FGDs, both among men and women. While some men felt sympathetic towards women’s covert use in certain circumstances, such as if they had many children already, the majority asserted that covert use could lead to loss of trust, separation, and even violence. Several men described how covert use threatened masculinity and relationships, particularly around fertility concerns:

*“It is very important because if a woman goes for family planning without informing me or consulting me, you know maybe as a man I expect one more child and I may be working very hard for that one and yet I don’t see it forthcoming, I might start to think that maybe I have a problem, it will bring worries and it may even bring conflicts.” (36 year-old man, 3 children).*

Concerns about contraceptive side effects created challenges for couple communication. One 48 year-old father of 5 described how his wife started using the injection without his knowledge, her sexual libido decreased, which led to relationship conflict. Another man, referring to prolonged bleeding while using the contraceptive implant, explained,

*“If you are not aware [of FP use], you might end up wasting resources thinking that your wife is sick but in real sense she is using family planning. And also if you are not aware you will go to chemists to get drugs…” (32 year-old man, 2 children).*

Most female participants shared men’s views that, ideally, the decision to start FP should be made in agreement with male partners. Discovering side effects during covert FP use was cited as a problematic issue in both FGDs:

*“It is important to sit down with your partner and agree if you want to start using family planning because if you do not agree it can cause problems…like when you start having side effects of family planning and he finds out it can even get physical.” (29 year-old woman, 2 children).*

Yet, women expressed that since women are ultimately responsible for FP and raising children, they have many strategies for negotiating FP use with partners, which may include covert use. Men have to be managed and “convinced”; as one 29 year-old mother of 2 put it, *“…it depends on how you package it and approach him—you must have a strategy.”* Only a few women said that if their male partners forbade them to use FP they would comply. For most female participants, initiating FP without their partners’ knowledge was a last resort, though anecdotes about the challenges of communicating with men were common.

### FP-related health concerns frame personal and community narratives

Both men and women cited concerns about contraceptive side effects and potential harms as the most prominent barrier to postpartum family planning uptake, and a major reason some men generally discourage contraceptive use. These concerns were interwoven throughout all FGDs, often unprompted. Women mentioned multiple concerns about FP-related side effects and harms, including excessive bleeding, weight gain, fatigue, and contraceptive failure. Among men, the most frequently discussed concerns were low female sexual desire, bleeding, future infertility, birth defects, hormonal imbalance, and inability to work. A few men asserted that very young women in particular should not use FP. Often, stories of contraceptive harms were used to frame personal and community narratives of FP. A male participant gave this example:

*“I am also seeing that side effects can make one fear going for FP. My friend…went for FP, bad luck she didn’t tell the husband, they have two [children] now but are looking for the third, they can’t get…So you see, that thing [FP] affected her. When she shared with another lady, through her experience the lady also dismissed FP to be bad.” (29 year-old man, 1 child).*

### Perspectives on receiving SMS about FP topics

Both men and women were asked about how they would feel about receiving FP-related SMS, whether and how men should be involved in a FP-oriented mHealth program, and preferred SMS topics. All men reported being open to receiving FP-related SMS, and discussed multiple advantages of using SMS dialogue for FP counseling, including: reaching hard-to-reach men with information, potential for anonymity when asking clinicians sensitive questions, convenience, and triggering improved communication with female partners. Several men asserted that repetitive messaging would be most effective at increasing awareness and knowledge among men. One male participant compared receiving FP-related messaging to advertisers’ success promoting a prominent toothpaste brand:

“I think frequent messaging can change people’s thinking about FP…The more these messages are received, the more people’s attitude will change positively.” *(29 year-old man, 1 child).*

Similarly, the repetition of SMS messaging was noted as a way to overcome negative ideas about FP among men in the community:

*“If a message gets into [your] phone but you never wanted to read it, when it keeps coming, you will have to react and read it. And this can change men’s attitude on talking about FP.” (28 year-old man, no children).*

In addition to repetition, most men felt that they needed the “truth” about FP, and SMS could be a vehicle for accurate information. In general, men distrusted female partners as sources of FP information, and wanted to avoid the influence of other community members in their FP decision-making. Speaking about ideal messaging content, one participant explained,

*“The messages that would help couples use FP as per my opinion: methods of FP, their side effects, advantages and disadvantages…so when I read the message then I can share with my wife, then we decide on which one of them is good with us. But when you send the messages, kindly tell us the truth of that family planning, OK, we know that there are some that cause hormonal imbalance…” (28 year-old man, no children).*

A few men were concerned that the sample messages were too long, and message length would deter male recipients from reading them. Another man noted his general response to FP-related SMS would depend on perceived appropriateness of message timing and content. One man put it this way:

*“[Men’s reaction will] depend on the information. Example, maybe you had an FP method for 4 or 5 years and that time has elapsed and you are being reminded that you are supposed to come back for FP that’s good but when you are planning to have a child and you get such kind of a message, I will react against it.” (27 year-old man, 1 child).*

With respect to preferred content and timing, women overwhelmingly preferred to receive information about FP during pregnancy, rather than waiting until after they had delivered, *“so that after giving birth I will know the method I am going to use” (19 year-old woman, 1 child).* Topics of interested included the benefits of FP, information about available FP methods, general encouragement to use FP, and antenatal/neonatal care. The ability to get real-time advice over SMS was seen as particularly valuable, as it could be used as a “short cut” to avoid unnecessary clinic visits and receive information from a reputable source—as opposed to being influenced by community members without appropriate expertise:

*“I think that the SMS is good, because let’s say you have been given Depo Provera and it is affecting you, so through the SMS you can be able to ask questions and get responses to any worries that you may have. It is better than asking someone from outside, they can give you wrong advice.” (19 year-old woman, 1 child).*

All but one female FGD participant felt that male partners should be included in an FP-related mHealth intervention using SMS. If men receive the same messages about FP as women do, participants asserted, they would be less suspicious of the information and intent. As one woman put it,

*“It [SMS] is a good idea because when you [clinicians] educate them they will listen and will not take it as if we are the ones who have come with the idea from outside to lie to them…so when you are the ones who have educated them at least they can listen to what we have to say about it and it can also make them accept use of family planning.” (29 year-old woman, 2 children).*

A few women felt that in order for SMS to be well-received by men, they should be primed with in-person FP education; others simply felt men must not be “surprised” by FP-related messaging, but should be told to expect it. Women in both FGDs were specifically asked about potential negative effects of men receiving messaging on FP, and the perceived impact on women’s decision-making or autonomy. Only one participant brought up a concern:

*“I think it will depend on an individual, they [SMS] can be sent to him and he will use the same information to try and convince you not to believe anything that the message says, because he believes that a married woman should not use family planning—she should be giving birth.” (20 year-old single woman, 2 children).*

A contradiction emerged with respect to men’s views on receiving information about potential FP side effects via SMS. Men wanted to hear both the positives and the negatives about FP for a balanced understanding: *“the messages should clearly demystify side effects to avoid blame later” (30 year-old man, 1 child).* Learning about potential contraceptive side effects was generally perceived as a way to dispel fear and misconceptions about FP:

*“What to say is maybe about these side effects. They [SMS] should talk about it clearly, in fact…I have seen some of the effects which are real but when you consult they tell you that it’s not true…So they should come out clear and tell us. That is something that makes us fear this family planning at times.” (36 year-old man, 3 children).*

While men’s preferences about receiving side effect information were clear, several reacted negatively to a sample SMS message about irregular bleeding being normal with the implant or injection (Please see Table 2, sample message 4). The message was thought likely to actually discourage FP use, for various reasons. Some men felt that anticipating bleeding, even if told it was normal and healthy, would induce fear of negative health effects. Another man said, *“The message is very discouraging… sometimes you want sex and this means you will not be doing it.” (25 year-old man, 1 child).*

**Table 2:** Examples of SMS messages used in FGDs to elicit feedback (English)

1. {name}, this is {nurse} from {clinic}. Over the next months we will send you messages to help you make family planning decisions. Spacing pregnancies is good for your health and your children’s health. Are you planning another pregnancy in the future?	
2. {name}, this is {nurse} from {clinic}. There are many family planning methods that are safe and effective while breastfeeding. Did you know that women can get pregnant even while they are breastfeeding?	
3. {name}, this is {nurse} from {clinic}. Now that your baby is born, it is important to think about planning your family in the future. Have you talked about family planning as a couple?	
4. {name}, this is {nurse} from {clinic}. While using Jadelle or the injection, it is common to have bleeding that comes and goes at any time. This bleeding is normal unless it is very heavy. Do you have concerns about bleeding?	
5. {name}, this is {nurse} from {clinic}. Many couples use condoms to prevent HIV and another family planning method at the same time to prevent pregnancy. Talk about using condoms as a couple.	

### SMS and couple communication

The majority of men and women in all FGDs felt that receiving SMS about FP could promote improved communication with their partners. Overall, male participants perceived that it was easier to communicate about FP within the couple “*when you already have the facts”,* in other words, when FP education had been sent in SMS form directly to their phones. Men felt that they should receive SMS alongside their partners, *“so that it [SMS] would make a point of discussion as a family” (26 year-old man, 2 children.)* Furthermore, a couples-oriented SMS program may dispel suspicion around women’s covert contraceptive use, allowing for discussion:

*“If both get the message, it will be better because if the wife gets it alone then the man can be suspicious that [she has] colluded with somebody so that [she] can go for FP, but if the man also gets it…they can discuss it peacefully.” (25 year-old man, 1 child).*

In response to reading sample SMS messages, a male participant described how receiving such messages could bring FP to the forefront of conversation within the relationship:

*“…When you get such a message you will have to talk about it after receiving the message and…I’ll talk about it with the wife, we will discuss it and if I didn’t have it in mind it will make me to have it in mind. Every time I get the message I think about that family planning.” (40 year-old man, 2 children).*

Women discussed that SMS, as well as other sources of FP education for men, would indeed make it easier to communicate with them about using contraception postpartum. The concept that the information had to come from someone outside the relationship was again central in both FGDs. For example, a 29 year-old woman said,

*“Sometimes discussing family planning with your husband can be hard because he doesn’t support it, so I think SMS can help; you just show him and tell him it is from the hospital.” (29 year-old partnered woman, 4 children).*

Apart from communication within the couple, several men felt that receiving FP-related SMS could make an impact with men in the larger community, rather than simply at the personal or dyadic level:

*“Our egos are too big to sit down to discuss women’s issues. So I think when you get a message you will go and tell a friend. You know I got a message and the message is about family planning, the friend will also tell you I got the same so from there you will begin to … realize that you demystify the myth that it’s something that only women should talk about.” (32 year-old man, 2 children).*

Similarly, FP-related SMS was perceived to have the potential to *“promote consultation [discussion among men], because when I get such a message, I will run to my friends and share with them the message…” (29 year-old man, 1 child).* On the other hand, a minority of men felt that receiving SMS would not be enough to change skeptical men’s minds about FP, and would not be as effective as an intervention involving human interaction, particularly with a clinician. A 25 year-old male participant argued,

*“Me I think there would be no change. What if I have a negative attitude towards FP, then I get such a message, that message will not convince me compared to how a human being or an expert would convince me.” (25 year-old man, 1 child).*

## Discussion

As the use of mHealth technology for SRH programming continues to gain traction across the Global South, this study provides insight into end-users’ perspectives on a theory-driven SMS approach to postpartum FP education and counseling. Additionally, our findings shed light on how men can and should be included in an FP-focused SMS dialogue intervention, and the anticipated effects of such an intervention on couple communication. These data informed the design of the Mobile WACh XY intervention and RCT, specifically relating to message timing and content, male inclusion, and system adaptation (Table 3). To our knowledge, this is the first published study to qualitatively examine the formal inclusion of men in a couples-oriented mHealth program for FP.

**Table 3:** Influence of formative findings on intervention design

*Message timing and content:*	
∙ The FGD findings supported initiating messaging during pregnancy rather than waiting until postpartum. ∘ *Weekly automated messages started at enrollment in the trial, which occurred during the third trimester of pregnancy (at/after 28 weeks).* ∘ *Automated messages stop at study exit at 6 months postpartum.* ∙ Some SMS were shortened in response to feedback from male participants. ∙ Despite divergent views from men on anticipatory guidance about contraceptive physiologic or side effects, especially irregular bleeding, these messages were included. ∘ *Perspectives on such anticipatory guidance will be explored further in post-RCT in-depth interviews with male and female intervention participants.* ∙ We developed tailored messaging tracks for individual women who were single/did not desire partner involvement and for couples.	
*Male engagement:*	
∙ While the FGD findings support the acceptability of including male partners, male partners must not be recruited without the express informed consent of the woman. ∙ Women who desired to consult with male partners prior to referring them for trial participation were given this option. ∙ Men received detailed information about the intervention and also signed informed consent prior to receiving SMS.	
*SMS platform adaptation:*	
∙ In response to the pervasive side effect concerns and misperceptions, framed with narratives of contraceptive discontinuation, the SMS platform was adapted to allow for method-specific messaging “tracks” after method choice. ∘ E.g.*: After a woman/couple choose the injection, subsequent messaging track is designed to support continuation through engagement around effects, adherence, and option to change methods.* ∘ *Method-specific tracks also eliminate non-personalized messages about other methods once a method has been initiated.* ∙ In consideration of promoting couple communication, a feature was added to the system to allow for forwarding of participant messages to his/her partner if appropriate. ∘ E.g.*: If a male partner sends in an SMS to the nurse requesting more information about the method, this SMS could be forwarded to the female partner, allowing both members to interact.* ∘ *This feature is not automatic; the clinician can decide whether it is appropriate to forward specific messages.*	

Men in this study strongly desired inclusion in FP education, decision-making, and programming, which is in line with other published studies from western Kenya [18, 31] and elsewhere in Africa [32–34]. They responded enthusiastically to the idea of receiving FP-related SMS along with their female partners, citing the benefits of repetition of FP information, SMS as a prompt for couple communication around FP, and the option of communicating directly with a provider.

However, male resistance to FP in the context of unequal relationship power dynamics may also be a contributor to women’s unmet need for contraception [35]. We recognize that male inclusion in an FP-related intervention may not be beneficial for all women or couples, and were particularly concerned with the possibility of harm to women. Yet, despite being asked specific questions about potential harms of including men, women raised very few concerns, and emphasized instead the need to educate men about FP in order to improve women’s FP access. However, women’s relative lack of privacy concerns in the current study contrasts the findings of another formative study conducted by our group among pregnant and postpartum women accessing HIV care, undertaken prior to initiating an mHealth intervention targeting Option B+ adherence and retention [29]. Women in that study alluded to the discovery of covert FP use and relationship conflict if male partners saw FP content on their phones, and suggested that some women would not want to receive SMS for this reason [36]. It is possible that SMS privacy concerns were more prominent among a population of women living with HIV due to concurrent concerns about inadvertent HIV disclosure with SMS [37]. Alternatively, though we specifically probed this topic, perspectives from the limited number of study participants may not reflect diverse views from other men and women, or participants may have felt social desirability bias in the group setting.

Men and women consistently identified the potential for FP-related SMS to act as a prompt, or trigger, for couple communication around FP. Other than the desire for accurate FP information, this was the most frequently-discussed benefit of a two-way SMS intervention. Challenging, or “problematic” [38] communication within couples around reproductive issues has long been highlighted as a contributor to unmet need for contraception in sub-Saharan Africa [35, 39–41], and reported communication around childbearing and FP within couples is associated with contraceptive use [16, 42]. A qualitative evaluation of the Malawi Male Motivator project, a community-based intervention to promote male involvement in FP, reported that participants experienced increased frequency of communication and shared decision-making with their spouses and were more likely to use contraception [43]. These data suggest that supporting couple communication may contribute to improved contraceptive access. While women’s covert contraceptive use was raised in all discussions, this strategy was generally viewed as a last resort among both women and men, to be used when couple communication fails or is not possible [18]. Corroborating other qualitative research among men in the region [31, 34, 43], participants in this study emphasized the need for FP information to be introduced by a trusted source from outside the couple, bypassing male distrust of women’s FP knowledge. This study contributes a perspective specific to an mHealth intervention, that SMS dialogue may present an innovative strategy to promote couple FP education and subsequently support couple decision-making. Thinking beyond the dyad, while the receipt of SMS messages on a personal phone is an individual- or couple-level intervention, men in our study felt that there could be a community-level effect through the sharing of SMS messages with male peers. More research is needed into what type of messaging is effective in promoting individual, couple, and community-level behavior change.

Contraceptive health and side effect concerns, often intermixed with myths and misinformation, were pervasive in participants’ discussions of FP decision-making. Numerous studies have observed similar concerns as key barriers to contraceptive use among women [44, 45], postpartum women [8, 10], and men [32, 46]. Indeed, fear of side effects or harms are the primary reason for contraceptive discontinuation in Kenya, accounting for 28–52% of hormonal method and intrauterine device discontinuation [47]. There is currently no data on whether FP education and real-time support via two-way SMS can improve continuation or change misperceptions about side effects. Yet, in light of the community presence of these concerns, approaches such as SMS that can reach people in the community rather than the facility setting only are needed [48, 49]. Incongruity existed between men’s strong desire for the “truth” about FP and the minority of men who felt SMS messages with anticipatory guidance about methods’ physiologic effects or side effects would actually deter FP use.

Our study has several limitations. Female participants were all accessing antenatal or postnatal healthcare, and the majority were married. It is possible that women in less stable partnerships or with poorer access to care are less likely to use FP, and may have different preferences and privacy concerns about receiving FP-related SMS. We recruited men via referral from female partners, and thus men who were sampled for/agreed to participate in this study may have been more open to FP discussion than men in the community not referred by women. FGDs were conducted at health facilities; participants may have associated moderators with clinicians and responded to questions in a way they thought would be socially desirable. Finally, FGDs were translated from DhoLuo, and nuanced meanings may have bene lost. While findings from our study may not directly generalizable to other settings, results contribute new perspectives on male inclusion in mHealth FP programming that may be applicable elsewhere.

## Conclusions

Postpartum unmet need for FP is unacceptably high. In light of social and health systems barriers to contraceptive access among postpartum women in resource-limited settings, innovative and tailored approaches are needed to more effectively reach this key population. Interactive mHealth interventions using SMS may be a useful tool for delivering postpartum FP education and counseling, and provide a novel approach to including male partners where appropriate. However, even in settings where male involvement is acceptable and desired, individual women should have the option to protect their own reproductive privacy and autonomy by opting out of couples-based SRH programming. Formative research is critical in the development of impactful and acceptable SMS content for mHealth interventions in SRH. Future studies should clarify whether receiving information about perceived side effects prior to and/or in response to using a method alters method initiation, continuation, and user satisfaction. Furthermore, additional research is needed to identify which messaging strategies are most effective for women, men, and couples, and to rigorously evaluate whether and how SMS programs improve FP outcomes.
